# Baseline Vitamin D Levels on Quality of Life and Pain Perception Among Patients with Chronic Pain with Long-Term Prescription Opioid Use: A Prospective Study

**DOI:** 10.3390/jcm14020645

**Published:** 2025-01-20

**Authors:** Gabija Laubner Sakalauskienė, Indrė Stražnickaitė, Sigutė Miškinytė, Linas Zdanavičius, Jūratė Šipylaitė, Robertas Badaras

**Affiliations:** 1 Clinic of Anaesthesiology and Intensive Care, Institute of Clinical Medicine, Faculty of Medicine, Vilnius University, LT-03101 Vilnius, Lithuania; jurate.sipylaite@mf.vu.lt (J.Š.); robertas.badaras@mf.vu.lt (R.B.); 2 Faculty of Medicine, Vilnius University, LT-03101 Vilnius, Lithuania; indre.straznickaite@gmail.com (I.S.); sigute.miskinyte@gmail.com (S.M.); linas.zdanavicius@gmail.com (L.Z.)

**Keywords:** chronic pain, vitamin D, prescription opioids, quality of life

## Abstract

**Objectives:** To investigate the correlation between baseline serum concentrations of 25-hydroxyvitamin D (25-OHD) and quality of life (QoL), as well as pain perception in patients with chronic pain with long-term prescription opioid usage before opioid detoxification. **Methods:** We prospectively studied 45 patients with chronic pain with long-term prescription opioid usage who were selected for elective detoxification. Baseline serum 25-OHD levels were measured prior to detoxification, classifying patients as either vitamin D deficient (<75 nmol/L) or sufficient (≥75 nmol/L). QoL was assessed using the SF-36v2^TM^ questionnaire, while pain levels were assessed using Visual Analogue Scale (VAS) scores before treatment. **Results:** Mean pain scores before detoxification of the patients with sufficient baseline 25-OHD levels vs. those with deficient levels were, respectively, 6.06 ± 2.32 vs. 6.86 ± 2.10 (normalized scores 1.22 ± 0.571 vs. 0.950 ± 0.632; *p* = 0.164). The analysis of SF-36v2™ questionnaire scores revealed minimal variation between groups (35.00 ± 14.198 vs. 34.97 ± 13.52), indicating no significant association between Vitamin D levels and QoL (*p* = 0.913). **Conclusions:** The analysis of baseline 25-OHD levels in relation to QoL assessments and pain scores did not reveal a statistically significant association, indicating that variations in baseline vitamin D levels may not substantially impact QoL or pain perception. Further studies may help determine how to assess and optimize vitamin D levels in patients with chronic pain on long-term prescription opioids.

## 1. Introduction

Although opioids have been historically recognized for their effectiveness in managing severe acute pain and chronic pain from advanced conditions [1], their long-term use is increasingly questioned due to significant risks and adverse effects [2]. Despite the opioid crisis that has emerged in the United States over the past decade and the growing global concerns regarding addiction and misuse, opioid analgesics are still commonly prescribed for the management of chronic pain [3]. However, their long-term efficacy has been called into question, with evidence indicating that their effectiveness is often surpassed by alternative treatments, such as non-opioid analgesics and interventional therapies [4]. However, prolonged opioid use is associated with significant adverse effects, including constipation, sleep-related breathing disorders, fractures, dysregulation of the hypothalamic–pituitary–adrenal axis, overdose, and an increased risk of addictive behaviors [2,5]. The development of tolerance and opioid-induced hyperalgesia further complicates the long-term clinical use of opioids [2,5].

In recent years, there has been a growing interest in the potential role of vitamin D in pain management. Vitamin D is known to regulate various physiological processes, including immune function, inflammation reduction, tumor suppression, antioxidant activity, and neuroprotection [6,7,8,9,10].

Low baseline levels of vitamin D have been associated with diminished physical functioning, increased pain sensitivity, and heightened fatigue, all contributing to a lower perceived quality of life, particularly in chronic pain patients [11]. Several studies have suggested a link between vitamin D levels and the occurrence of both acute and chronic pain [12,13,14,15].

Patients with chronic pain frequently exhibit insufficient or deficient levels of vitamin D, with research indicating a link between lower vitamin D levels and increased pain severity [15,16,17]. Deficient vitamin D levels are associated with poorer health-related quality of life, potentially due to heightened central sensitivity and augmented pain response to mechanical stimulation; however, vitamin D appears less directly associated with the experience of spontaneous chronic pain as reported by patients [11].

Additionally, there is evidence to suggest that reduced levels of 25-OHD are associated with increased opioid consumption, raising the possibility that vitamin D deficiency may exacerbate opioid tolerance [8,18,19].

Despite these associations, the exact physiological relationship between vitamin D deficiency and pain genesis remains ambiguous [16]. While some studies have suggested that vitamin D supplementation may alleviate pain symptoms and QoL, the evidence is inconsistent.

This study aimed to investigate the correlation between baseline serum concentrations of 25-OHD and QoL and pain perception in patients with chronic pain recruited for opioid detoxification after long-term prescription opioid use. The findings are preliminary and based on a relatively small sample size; thus, further research with larger cohorts is needed to establish definitive conclusions regarding the relationship between serum 25-OHD levels, QoL, and pain perception.

## 2. Materials and Methods

The methodology applied in this study is an extension of the approaches utilized in our earlier research [20], which examined the impact of prescription opioid detoxification on quality of life and pain levels.

This prospective study, carried out from 2019 to 2023, involved a group of 45 patients recruited for detoxification from prescription opioids at the Toxicology Centre of Republican Vilnius University Hospital, Lithuania. The study was approved by the Vilnius Regional Committee on Biomedical Research Ethics (approval no. 2019/10-1153-644), and all participants provided written informed consent prior to inclusion. Referrals to the Toxicology Centre were made by both primary care physicians and secondary care providers, including the National Centre for Cancer and Pain Clinics, where patients were assessed for their eligibility for detoxification from prescription opioids.

Participants were required to meet the following criteria for inclusion in the study: adult patients with documented long-term prescription opioid usage for the management of chronic pain, documented opioid tolerance, confirmed dependence on prescription opioids, and admission to the Toxicology Centre for elective detoxification. Opioid tolerance was defined as the need for progressively higher doses to achieve effective pain relief due to a diminished analgesic response. Exclusion criteria ruled out individuals with acute opioid poisoning, diagnosed addiction to illicit opioids, or addiction to multiple psychoactive substances.

Serum levels of 25-hydroxyvitamin D (25-OHD) were measured in an ambulatory setting prior to the initiation of detoxification. Patients with baseline serum 25-OHD levels below 75 nmol/L were classified as having deficient vitamin D levels, while those with levels of 75 nmol/L or above were categorized as having sufficient vitamin D levels.

It is important to note that the definitions of vitamin D deficiency, insufficiency, and optimal serum levels vary within the medical literature. In 2021, the Lithuanian College of Family Physicians published “Guidelines for the Diagnosis, Prevention, and Treatment of Vitamin D Deficiency”, which recommended a range of 100–150 nmol/L as the optimal serum concentration for 25-OHD, with levels between 75–100 nmol/L being considered suboptimal [21]. The European Food Safety Authority declares 50 nmol/L to be an appropriate target value for serum 25-OHD concentration [22]. The International Society of Endocrinology, in their “Guidelines for the Evaluation, Treatment, and Prevention of Vitamin D”, deemed 75 nmol/L to be the normal value for serum 25-OHD levels [23]. This criterion was adopted as the cut-off point in our study. The specific definition of appropriate serum 25-OHD levels remains a matter of ongoing debate and necessitates individualized determination within the pertinent clinical context [24].

Upon admission, eligible clinical and demographic information was gathered. This information encompassed details such as the patient’s age, the duration of their pain, their primary pain location or diagnosis, their opioid consumption, and the length of time they had been using opioids. The opioid dosage at admission was ascertained through a combination of patient self-report and a review of their medical records. For ease of analysis, the opioid intake was subsequently converted into oral morphine equivalents (MEDs).

The patient cohort consisted of individuals prescribed opioids primarily for managing various pain conditions, including headaches, cancer-related pain, back pain, rheumatoid arthritis, gastrointestinal pathology, chronic muscle pain, and arthrosis of the humerus. Notably, all cancer patients included in the study were in the remission phase and not undergoing active cancer treatment at the time of data collection.

This detoxification protocol was developed based on our previous research [25].

QoL was assessed using the SF-36v2™ questionnaire in the Lithuanian language. Patient pain intensity was measured with the Visual Analogue Score (VAS). VAS used in this study ranged from 0 to 10, where 0 indicated no pain and 10 represented the worst pain imaginable (Table 1).

It is important to note that throughout the broader study [20], of which this sub-study is a component, QoL and VAS pain scores were systematically measured both before and after the detoxification process. However, for the purposes of this specific analysis, we exclusively collected and analyzed data from QoL SF-36v2™ questionnaires and VAS pain scores recorded prior to detoxification and compared these with serum 25-OHD levels in two distinct groups, classified according to baseline vitamin D status: one group with deficient levels and another with sufficient levels. This approach was chosen to evaluate the relationship between pre-detoxification QoL, pain perception, and vitamin D status.

On the first day of detoxification, patients’ 25-OHD levels were modulated through vitamin D supplementation, specifically by administering a single dose of 50,000 IU of cholecalciferol to those with deficient baseline serum 25-OHD levels. The selection of formulation, specifically the oral solution, and the prescribed dosing regimen adhered to the recommendations outlined in the Lithuanian College of Family Physicians Guidelines for the management of vitamin D deficiency [20]. Upon discharge, supplementation and continued care under a family physician were recommended for all patients. It is noteworthy that 25-OHD levels were not measured after inpatient detoxification, as the study primarily focused on the influence of baseline 25-OHD levels on pre-detoxification QoL and pain perception.

In this study, we tested the null hypothesis that there is no significant association between baseline vitamin D levels, pre-detoxification QoL, and VAS pain scores.

The analysis of data was conducted utilizing MS Excel and IBM SPSS 23.0 software. Continuous data were presented as the mean with accompanying standard deviation, while proportions were expressed in percentages.

To assess the relationships between 25-OHD levels, VAS pain scores, and QoL scores before opioid detoxification, we performed a linear regression analysis using ordinary least squares (OLS) estimation. In this model, 25-OHD levels (log-transformed to correct for skewness and enhance linearity) and VAS pain scores before detoxification (reversed and log-transformed for similar reasons) were included as independent variables, while the QoL score served as the dependent variable. Statistical significance was evaluated at the 0.05 level, with corresponding t-statistics and *p*-values indicating the significance of each predictor’s contribution to the model.

Additionally, an independent samples t-test was conducted to compare differences between two groups: participants with sufficient 25-OHD levels (≥75 nmol/L) and those with deficient levels (<75 nmol/L). This test, assuming unequal variances, assessed mean differences across key variables—VAS pain score before intervention (reversed and log-transformed) and QoL SF-36v2™ questionnaire responses—between the groups. Results were reported with mean differences, standard errors of the differences, and significance assessed at the 0.05 level.

This approach ensured that transformations were applied appropriately to normalize data distributions and enhance the interpretability of relationships in both the regression model and *t*-test comparisons.

## 3. Results

The results presented are based on data from a cohort of 45 patients, with 28 women, constituting 62.22% of the cohort. The average age of the participants was 53.62 ± 12.70 years. Participants had a history of prescribed opioid usage spanning an average of 60.51 ± 67.81 months, and the prescribed dose of opioids averaged 139.8 ± 153.9 mg per day in oral morphine equivalent doses (MEDs) (Table 2).

The use of opioids in the patient cohort was predominantly for the management of headaches and cancer, with each accounting for 14 out of 45 cases. Back pain was the next most common reason, reported in 10 out of 45 cases. Other, less frequent indications included rheumatoid arthritis and gastrointestinal pathology, each representing 2 out of 45 cases. Additional conditions for which opioids were prescribed, though in even smaller numbers, were post-burn sequel, chronic muscle pain, and arthrosis of the humerus, each affecting 1 out of 45 cases (Table 2).

The prescription data indicate a variety of opioids being utilized within this group. Tramadol emerged as the most prescribed opioid, with 13 patients receiving it, followed by codeine and morphine, each prescribed to 9 patients. Other opioids prescribed less frequently included fentanyl in its transdermal form to four patients, pethidine to two patients, and methadone and oxycodone to one patient each. Notably, some patients were prescribed multiple opioids: two patients received both morphine and fentanyl, another two were prescribed fentanyl and tramadol, one patient received a combination of morphine and tramadol, and one patient was prescribed codeine, morphine, and tramadol concurrently.

**Figure 1:**
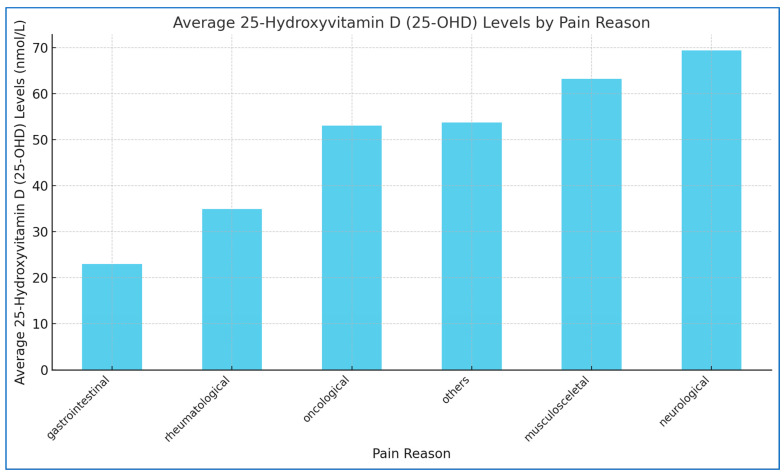
Average 25-hydroxyvitamin D (25-OHD) levels by pain reason.

The mean serum 25-OHD concentration across the study cohort was 58.3 ± 35.2 nmol/L. Among participants, those with serum 25-OHD levels ≥75 nmol/L (n = 16) had a mean concentration of 98.39 ± 28.04 nmol/L, whereas individuals with levels <75 nmol/L (n = 29) exhibited a mean concentration of 38.26 ± 17.22 nmol/L (see Table 2). Notably, 64.4% of the cohort fell into the deficient category, defined by serum 25-OHD levels below the 75 nmol/L threshold. Figure 1 illustrates the average 25-Hydroxyvitamin D (25-OHD) levels (nmol/L) across different pain-related conditions.

### 3.1. Association of 25-OHD Levels, Pain Scores, and Quality of Life: Regression Findings

A linear regression analysis was conducted to evaluate the relationship between 25-OHD levels (log-transformed), VAS pain scores before opioid detoxification (reversed and log-transformed), and QoL scores. The model fit measures indicated a low correlation coefficient (R = 0.123) and an R-squared value (R^2^) of 0.0150, suggesting that only 1.5% of the variance in QoL SF-36v2™ questionnaire scores can be explained by the predictors in the model.

The intercept was statistically significant (β = 35.32, SE = 13.37, t = 2.642, *p* = 0.012), indicating that the baseline QoL score was significantly different from zero when the predictor variables were at their reference values. However, the coefficients for 25-OHD levels (β = −1.79, SE = 7.88, t = −0.227, *p* = 0.821) and VAS pain scores (β = 2.73, SE = 3.42, t = 0.796, *p* = 0.430) were not statistically significant, suggesting no meaningful relationships between these variables and QoL scores.

### 3.2. Association Between Serum 25-OHD Levels and QoL SF-36v2™ Questionnaires

**Figure 2:**
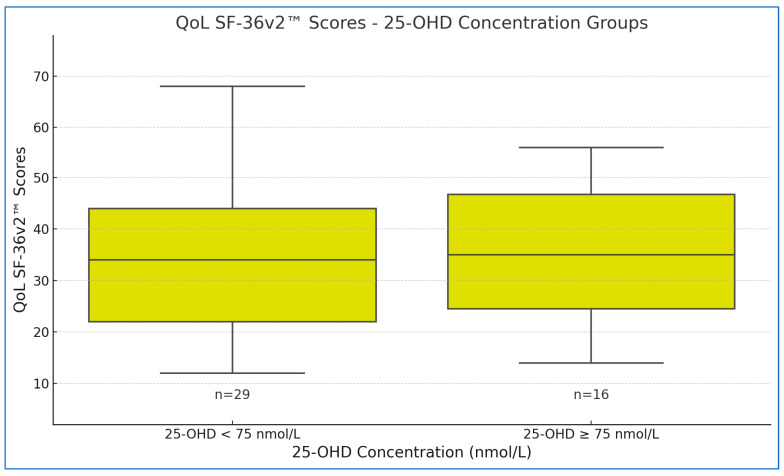
The association between 25-hydroxyvitamin D (25-OHD) levels and quality of life (QoL) as measured by the SF-36v2™ Questionnaire score.

The independent samples t-test results indicated no statistically significant differences in QoL between participants with sufficient and deficient 25-OHD levels. Specifically, no significant differences were observed in SF-36v2™ questionnaire responses related to QoL (t (43) = 0.110, *p* = 0.913). The mean difference was 0.472 (SE = 4.286), with the sufficient vitamin D group scoring a mean of 35.44 (SD = 14.198) and the deficient group a mean of 34.97 (SD = 13.524). These findings suggest that vitamin D sufficiency did not significantly impact QoL scores in this sample (Figure 2).

### 3.3. Association Between Serum 25OHD Levels and VAS Pain Scores

An independent samples t-test was conducted to evaluate differences in VAS pain scores between two groups based on serum 25-OHD levels (≥75 nmol/L vs. <75 nmol/L). Participants with serum 25-OHD levels ≥75 nmol/L (n = 16) had a mean VAS pain score of 6.06 ± 2.32, while those with levels <75 nmol/L (n = 29) had a mean score of 6.86 ± 2.10. (Table 2). The normalized scores for the groups were 1.22 ± 0.571 and 0.950 ± 0.632, respectively.

**Figure 3:**
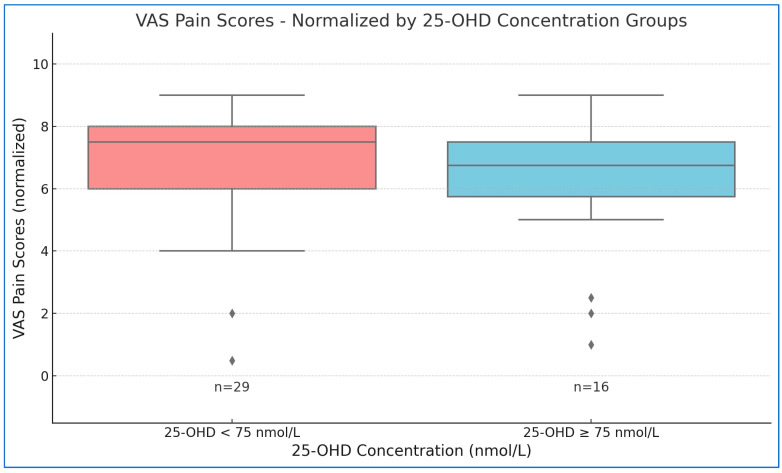
The association between 25-hydroxyvitamin D (25-OHD) levels and pain intensity.

The t-test results indicated no statistically significant difference in VAS pain scores between the two groups (t (43) = 1.415, *p* = 0.164), with a mean difference of 0.269 (SE = 0.190). This suggests that, although the group with sufficient vitamin D levels had a marginally higher mean VAS score (M = 1.22, SD = 0.571) compared to the deficient group (M = 0.950, SD = 0.632), this difference was not statistically significant, indicating no substantial effect of vitamin D levels on pain scores in this cohort (Figure 3).

## 4. Discussion

Vitamin D insufficiency and deficiency are recognized as global health concerns. Epidemiological data indicate that approximately 40 percent of adults in the USA have inadequate serum vitamin D levels, with vitamin D deficiency rates ranging from 13 to 60 percent across different regions of Europe [26,27,28]. Poor vitamin D levels are also prevalent in Lithuania, with reports suggesting that up to 70 percent of Lithuanian adults have inadequate vitamin D levels [29,30]. The findings of this study are consistent with these trends, as 64.4 percent of participants exhibited 25-OHD levels below the normal range.

Although previous research has suggested a link between low 25-OHD levels and the incidence of both acute and chronic pain [12,13,14,15], the current study did not establish a statistically significant association between serum 25-OHD levels and pain scores. This contrasts with earlier studies that have reported a positive impact of vitamin D supplementation on reducing pain, including improvements in VAS scores and reductions in opioid dosages for palliative cancer patients [18,31,32,33,34]. However, the absence of significant findings in this study highlights the complexity of the relationship between vitamin D and pain perception [12,13,16].

According to WHO guidelines, mild to moderate pain can be managed without opioid analgesics or with weak opioids such as codeine [35]. While the potential improvement in VAS scores through vitamin D supplementation could be beneficial for individuals with chronic pain and for healthcare providers [31,32,33,34], our study did not demonstrate a significant relationship between baseline vitamin D levels and pain scores. Although it is conceivable to hypothesize that vitamin D supplementation during detoxification might influence pain scores, the absence of significant differences in pain outcomes, combined with the lack of post-detoxification vitamin D measurements—attributable to the gradual nature of vitamin D metabolism [36] and the relatively short detoxification period, during which any fluctuations in 25-OHD levels were unlikely to result in significant long-term changes—limits our ability to draw definitive conclusions.

Research on vitamin D’s effect on QoL in chronic pain patients reveals mixed outcomes, with some studies indicating potential improvements in physical and psychological well-being through supplementation [37,38], while others report negligible effects and suggest that baseline vitamin D levels alone may not significantly influence QoL without considering concurrent factors, such as overall health status and pain levels [39]. Moreover, evidence suggests that routine supplementation may exert only a limited effect on QoL, with any observed benefits primarily confined to short-term studies within clinical populations [40].

Additionally, previous literature suggests that any QoL improvement due to vitamin D supplementation may require prolonged and sustained intervention, which may not be feasible or observable within a short detoxification timeframe [41]. As such, the current study may not fully capture the potential longitudinal effects of vitamin D on QoL, which could be more evident in studies with extended follow-up and comprehensive vitamin D monitoring.

Our study did not find a statistically significant relationship between baseline 25-OHD levels and QoL scores among participants, aligning with research suggesting that QoL outcomes in chronic pain patients are often influenced by multifactorial elements beyond vitamin D alone [40]. The absence of a clear association between 25-OHD levels and QoL in this cohort could reflect those factors inherent to opioid dependency and detoxification—such as physical withdrawal symptoms, emotional stressors, and social support levels—may play a more substantial role in determining QoL during this period.

The findings from this study highlight a limited association between serum 25-OHD levels, pain scores, and QoL outcomes in individuals undergoing opioid detoxification. The linear regression analysis demonstrated that 25-OHD levels and baseline VAS pain scores explained only a minimal portion (1.5%) of the variance in QoL scores, with no statistically significant coefficients for either predictor. This suggests that neither vitamin D levels nor initial pain severity substantially influences QoL outcomes in this sample, indicating the presence of other, more impactful factors on QoL during detoxification.

Further, no significant differences in QoL were observed between participants with sufficient and deficient 25-OHD levels, as indicated by independent samples t-tests. Both groups had comparable scores on the SF-36v2™ questionnaire, reinforcing that vitamin D sufficiency, defined by a 75 nmol/L threshold, did not markedly influence perceived QoL in this cohort.

While vitamin D levels were examined as a potential modulator of pain perception, our findings do not establish a conclusive link between baseline 25-OHD levels and pain perception in patients before opioid detoxification, despite a marginally higher mean pain score observed in those with sufficient 25-OHD levels. This result aligns with emerging literature that questions the direct role of vitamin D in pain modulation, particularly in chronic pain or opioid-dependent populations.

Despite these limitations, we believe that this analysis offers valuable preliminary insights, especially given the novelty of investigating the relationship between baseline vitamin D levels and pain perception in patients with chronic pain prior to opioid detoxification. While the results were not statistically significant, they revealed an intriguing trend that merits further investigation with a larger sample size.

This study, along with previous research of which it is a part [20], suggests that opioid cessation rather than baseline vitamin D levels plays a central role in influencing QoL and pain perception during detoxification. While focused on baseline serum 25-OHD levels in relation to pre-detoxification QoL and pain, future studies should assess 25-OHD levels both pre- and post-detoxification, using larger sample sizes or alternative approaches to better explore potential associations.

These findings highlight the complex factors influencing QoL and pain in individuals undergoing detoxification, suggesting that factors beyond baseline vitamin D levels may play a more substantial role. Future research should explore alternative biological, psychological, or social determinants, ideally using larger samples to better understand vitamin D’s impact on QoL and pain management. Further studies are also needed to identify factors that influence pain perception and assess whether adjunctive therapies, such as vitamin D supplementation, could benefit specific subgroups. Nonetheless, these preliminary results support opioid detoxification as a viable strategy for meaningful pain relief in opioid-dependent individuals, independent of baseline vitamin D status.

Future research should consider these multidimensional influences and employ larger sample sizes to more fully elucidate the role of vitamin D in QoL and pain management.

## 5. Limitations

This study acknowledges several important limitations. The relatively small sample size of 45 patients may limit the generalizability of the findings. Conducting the study at a single institution raises further concerns about applicability to other settings, particularly given local variations in vitamin D levels.

The lack of post-detoxification measurements of serum 25-OHD restricts the ability to assess changes in vitamin D status and its impact on pain outcomes throughout detoxification. While the reliance on the VAS introduces some subjectivity, it remains a widely accepted and validated measure of pain intensity, indicating that its use, despite potential limitations, is still appropriate for capturing pain experiences in this study.

Variations in definitions of vitamin D deficiency and sufficiency across guidelines complicate the interpretation of 25-OHD levels, highlighting the need for standardization in future research. Although potential confounding variables such as comorbidities and specific opioid types were not considered, the decision to refrain from stratifying participants by pain type or opioid type was intentional, ensuring adequate sample size and statistical power to enhance the robustness of the findings.

In conclusion, while this study contributes valuable preliminary insights into the relationship between baseline vitamin D levels, QoL, and pain in patients before opioid detoxification, these limitations underscore the need for further research to clarify these associations and enhance our understanding of effective pain management strategies in this population.

## 6. Conclusions

While baseline vitamin D levels were hypothesized to modulate pain perception and QoL in patients with chronic pain and long-term prescription opioid usage, our preliminary findings did not establish a definitive correlation between baseline 25-OHD levels, QoL, and pain perception.

These results suggest that opioid cessation may be the primary factor influencing pain perception and QoL, rather than baseline vitamin D levels. However, we observed a numerical trend indicating lower pain scores with higher 25-OHD levels, which suggests the possibility of a relationship that warrants further exploration. Although the current findings lack statistical significance, they indicate the importance of evaluating and potentially adjusting vitamin D levels in patients with chronic pain on prescription opioids, recognizing that individual responses can vary and that the benefits of optimal vitamin D should not be overlooked.

To better understand the potential relationship between vitamin D and pain management, further research is essential, focusing on larger sample sizes and more diverse cohorts, as well as incorporating post-detoxification vitamin D assessments and varied methodological approaches. This comprehensive strategy will yield more conclusive evidence and valuable insights into how baseline vitamin D levels influence QoL and pain perception in opioid-tolerant individuals. Thus, this current study serves as a preliminary step in exploring these complex relationships and emphasizes the need for continued investigation in this area.

## Figures and Tables

**Table 1:** VAS scoring criterion.

VAS Score	Pain Description
0	No pain
1–3	Mild pain
4–6	Moderate pain
7–9	Severe pain
10	Worst pain imaginable

**Table 2:** Patient characteristics.

Parameter	Value
Average Duration of Opioid Use	60.51 ± 67.81 months
Opioid Dose (mg/day, MEDs)	139.8 ± 153.9
Reason for Opioid Use (n, %)	Oncological (remission phase): 14 (31.1%)
	Neurological: 14 (31.1%)
	Musculoskeletal: 10 (22.2%)
	Rheumatoid: 2 (4.44%)
	Gastrointestinal: 2 (4.44%)
	Other: 3 (6.67%)
VAS by Condition (Mean ± SD)	Oncological (remission phase): 5.9 ± 2.6
	Neurological: 6.1 ± 1.6
	Musculoskeletal: 7.7 ± 2.1
	Rheumatoid: 6.8 ± 1.1
	Gastrointestinal: 8.5 ± 0.7
	Other: 8.5 ± 0.4
25-OHD Levels (Mean nmol/L ± SD)	≥75 nmol/L (n = 16): (98.4 ± 28.0)
	<75 nmol/L (n = 29): (38.3 ± 17.2)
25-OHD and QoL (percentage ± SD)	≥75 nmol/L (n = 16): 35.00 ± 14.198
	<75 nmol/L (n = 29): 34.96 ± 13.52
25-OHD and VAS (points ± SD)	≥75 nmol/L (n = 16): 6.06 ± 2.32
	<75 nmol/L (n = 29): 6.86 ± 2.10
Medications (n, %)	Tramadol: 13 (28.9%)
	Codeine: 9 (20%)
	Morphine: 9 (20%)
	Fentanyl: 4 (8.9%)
	Pethidine: 2 (4.4%)
	Methadone: 1 (2.2%)
	Oxycodone: 1 (2.2%)
Combination Use (n, %)	Morphine + Fentanyl: 2 (4.5%)
	Fentanyl + Tramadol: 2 (4.5%)
	Morphine + Tramadol: 1 (2.3%)
	Codeine + Morphine + Tramadol: 1 (2.3%)

## Data Availability

The original contributions presented in the study are included in the article material, and further inquiries can be directed to the corresponding author.
